# GAS6 signaling tempers Th17 development in patients with multiple sclerosis and helminth infection

**DOI:** 10.1371/journal.ppat.1009176

**Published:** 2020-12-21

**Authors:** Juan M. Ortiz Wilczyñski, Cinthia M. Olexen, Andrea E. Errasti, Mirta Schattner, Carla V. Rothlin, Jorge Correale, Eugenio A. Carrera Silva

**Affiliations:** 1 Institute of Experimental Medicine, CONICET-National Academy of Medicine, Buenos Aires, Argentina; 2 Institute of Pharmacology, School of Medicine, University of Buenos Aires, Buenos Aires, Argentina; 3 Department of Immunobiology and Pharmacology, Yale University, Connecticut, United States of America; 4 Department of Neurology, Fleni, Buenos Aires, Argentina; University of Dundee, UNITED KINGDOM

## Abstract

Multiple sclerosis (MS) is a highly disabling neurodegenerative autoimmune condition in which an unbalanced immune response plays a critical role. Although the mechanisms remain poorly defined, helminth infections are known to modulate the severity and progression of chronic inflammatory diseases. The tyrosine kinase receptors TYRO3, AXL, and MERTK (TAM) have been described as inhibitors of the immune response in various inflammatory settings. We show here that patients with concurrent natural helminth infections and MS condition (HIMS) had an increased expression of the negative regulatory TAM receptors in antigen-presenting cells and their agonist GAS6 in circulating CD11b^high^ and CD4^+^ T cells compared to patients with only MS. The Th17 subset was reduced in patients with HIMS with a subsequent downregulation of its pathogenic genetic program. Moreover, these CD4^+^ T cells promoted lower levels of the co-stimulatory molecules CD80, CD86, and CD40 on dendritic cells compared with CD4^+^ T cells from patients with MS, an effect that was GAS6-dependent. IL-10^+^ cells from patients with HIMS showed higher GAS6 expression levels than Th17 cells, and inhibition of phosphatidylserine/GAS6 binding led to an expansion of Th17 effector genes. The addition of GAS6 on activated CD4^+^ T cells from patients with MS restrains the Th17 gene expression signature. This cohort of patients with HIMS unravels a promising regulatory mechanism to dampen the Th17 inflammatory response in autoimmunity.

## Author summary

Helminths have co-evolved with human civilization, and the rapid exclusion from their environment, in the last few decades, has tremendously affected the immune development and regulation. Moreover, several epidemiological studies have shown an inverse correlation between the exposure of these organisms and the development of autoimmunity in industrialized countries. In this sense, helminth therapy appears to be a promising concept to oppose chronic inflammatory and autoimmune diseases because they are master manipulators of host immunity, albeit the mechanisms remain poorly defined. For this reason, it is essential to decipher the main regulatory pathways to hijack the immune response in the absence of parasite infection. Our research described how helminth infection promotes regulatory mechanisms based on the tyrosine kinase TYRO3, AXL, MERTK (TAM) receptors, and their ligand GAS6 to dampen Th17 development and the inflammatory response in patients with multiple sclerosis (MS), a neurodegenerative autoimmune disorder. We show here that GAS6 plays a critical role in the regulation of pro-inflammatory cytokines, transcriptional programs, and plasticity of IL-17 subset. Our work substantiates the hypothesis that enhancing the TAM axis in a manner similar to helminth infection could be a promising alternative for autoimmune diseases.

## Introduction

Multiple sclerosis (MS) is a chronic demyelinating and neurodegenerative autoimmune disease of the central nervous system (CNS) that frequently begins between 20 and 40 years of age. MS affects an estimated 2.5 million people worldwide and represents the second most common cause of nervous system disability in young adults after traumatic brain injury. The etiology of MS is complex and comprises both genetic and environmental risk factors. A strong correlation exists with certain HLA II alleles (mainly HLA-DRB1*15:01 and DRB5*01:01), pointing directly to CD4^+^ T cells as key players in MS [1–3]. Evidence shows that both Th1 and Th17 subsets are necessary for both the onset and progression of the disease. Th1 and Th17 cells mediate their pathogenic effect through a robust secretion of pro-inflammatory cytokines (eg, IL-17, IFN-γ, GM-CSF, TNF-α), which in turn activates resident microglia, astrocytes, infiltrating macrophages, and dendritic cells (DCs) recruited from the periphery, promoting a pro-inflammatory *milieu* with the synthesis of cytotoxic reactive oxygen and nitrogen species (ROS and RNS) [4–6]. One of the most striking illustrations of the importance of the environment in MS pathogenesis is its geographic distribution; prevalence rates are increased in high latitude regions yet uncommon near the equator [7]. Furthermore, individuals who move from a low-risk to a high-risk area, especially before the age of 15, show similar incidences to host country populations, suggesting the presence of either a protective factor in the region of origin or a harmful factor in the adopted region [8]. During the last century in developed countries, a significant increase in autoimmune and allergic diseases has been observed [9] that cannot be attributed to significant genetic alterations, implying a critical environment change occurred in this period. Candidates likely to be responsible for this effect in MS, alone or in combination with others, include sunlight-UV exposure with or without Vitamin D deficiency, viral infections, and cigarette smoking. Factors may not only influence disease onset at any time in the life of an individual but also affect relapse rates in patients presenting relapsing-remitting forms of MS [10].

The originally formulated “hygiene hypothesis,” later reformulated as the “old friends hypothesis,” establishes the necessity of the development of a properly “educated” immune system during our early years that is allowed by challenges from an array of harmless microbes [11,12]. Although many helminths could be harmful to their hosts in high burden, the longstanding helminth-host coexistence supports the concept that evolutionary forces shaped a balanced immune relationship in the context of Th2 response controlling also associated chronic inflammatory responses [13–16]. The type 2 immunity elicited against helminth tends to limit parasite burden and tissue damage, but also primes the immune response with regulatory programs [17]. In the last few decades, the incidence of helminth and bacterial infections has abruptly dropped in industrialized countries, and multiple epidemiological studies have shown an inverse correlation between the exposure of these organisms and the development of autoimmunity, bringing helminthic immunology under the spotlight [9,18–20]. Correale et al reported that natural helminth infections protect the course of MS in humans as evidenced by a reduction in the number of relapses, minimal changes in disability scores, and a significant decrease in the number of new or enlarging T2 lesions and gadolinium-enhancing lesions compared with uninfected individuals with MS [21]. The protection conferred by parasite infection was associated with IL-10 and TGF-β secretion, the increased suppressive activity of CD4^+^CD25^+^FOXP3^+^ T cells mediated by cell contact, and the induction of B regulatory cells [21,22]. Patients treated with anthelmintic drugs presented a significant increase both in the number of relapses and disability scores [23].

Considering the critical role of negative regulatory pathways in avoiding chronic inflammation and autoimmunity, we focus on a subfamily of receptor tyrosine kinases (TKR) that involves TYRO3, AXL, and MERTK (TAM) [24]. These receptors showed a preponderant role in innate immune response and their genetic ablation leads to an impaired immune response with exacerbated autoimmune features [25]. The engagement of these receptors by their ligands, PROS1 and GAS6, limit the magnitude and time course of the inflammatory response that can ensure a return to the baseline [26,27]. The endogenous agonists, PROS1 and GAS6, are Gla domain-containing proteins capable of binding exposed phosphatidylserine (PtdSer) in a Ca2^++^-dependent manner. We have previously shown that T cells activation induces PROS1 and externalizes PtdSer, thereby triggering TAM receptors on dendritic cells (DCs) to dampen the inflammatory response [27]. Recently, we have also elucidated that TYRO3 and PROS1 are key regulators of type 2 immunity in mouse models of allergy or helminth (*Nippostrongilus brasiliensis*) infection [28]. Finally, Nassar et al highlighted the essential role of GAS6 promoting tolerance in oral mucosa, as its absence leads to an imbalance in Th17 and Treg cells [29]. In the present work, we uncovered that helminth infections differentially modulate the expression of TAM receptors and ligands, and consequently the balance of Th17 response in patients with MS. We discovered that CD4^+^ T cells from patients with MS and concurrent natural helminth infections (HIMS) contained a lower percentage of Th17 cells as well as lineage-specific related genes and dampened DCs activation in a GAS6-dependent manner. This regulatory pathway could have important implications for new therapeutic alternatives.

## Material and methods

### Ethics statement

This study was approved by the Institutional Ethics Committee of the Raul Carrea Institute for Neurological Research (FLENI) and the Institutional Ethics Committee of the National Academy of Medicine (IMEX-ANM). All study participants provided written informed consent for the collection of blood samples and subsequent sample analysis.

### Patients and sample collection

The present study included 3 clinical groups, 31 healthy volunteers (HC, healthy control), 29 patients with multiple sclerosis (MS), and 18 patients with chronic helminth-infection and diagnosis of clinically definite relapsing-remitting MS criteria according to Poser’s or McDonald criteria [30,31]. Patients were recruited from a larger cohort of 1732 regularly followed patients with MS and were typical in all respects with the exception of eosinophilia. Any patient with MS with a high eosinophil count was included in the study. MS diagnosis preceded parasitic intestinal infection diagnosis by 38.3 + 7.3 months (range 18–45 months). Helminth-infected multiple sclerosis patients (HIMS) were confirmed by stool examination, eosinophilia, and IgE levels. Fecal samples were analyzed for parasite eggs, species identification, and the number of eggs per gram of feces, prepared by formalin-ether sedimentation. The HIMS subjects were infected with *Strongyloides stercolaris*, *Hymenolepis nana*, *Enterobius vermicularis*, *Ancylostoma duodenale*, *Ascaris lumbricoides*, *or Trichuris trichura* without undergoing anti-parasitic treatment at the starting point of the study. The presence of other endemic parasitoses, including trypanosomiasis, leishmaniasis, amebiasis, giardiasis, and toxoplasmosis, were excluded using microscopic examination and serological testing. In all parasite-infected patients with MS, the eosinophilia was not present during the previous two years. Five of the parasite-infected patients with MS enrolled here were previously reported [21].

Both eosinophil counts and stool examinations examined in uninfected MS subjects were found to be negative at study entry and throughout the entire study. Blood collection was performed during the remission phase of the disease. Blood draws were performed at least 90 days after a relapse or a course of steroids. No significant differences in age or gender proportion were observed among groups (**Table 1**). The EDSS score was significant lower in patients with HIMS (0.33 ± 0.188) compared with patients with MS(1.75 ± 0.185, p<0.0001) and all patients with MS and around the half of those with HIMS were under IFN-β treatment (**Table 1**).

**Table 1:** Clinical characteristics of the cohorts.

Characteristic	Control (healthy donor) n = 31	MS n = 29	HIMS n = 18
**Age (yr)** Mean +/- SD	36.7 ± 9.3	40.1 ± 7.4	38.5 ± 3.8
**Gender, n (%)** Men Women	12 (38.7) 19 (61.3)	10 (34.5) 19 (65.5)	6 (33.3) 12 (66.7)
**Annual relapse rate** 2 years before entry	NA	0.83 + 0.40	0.80 + 0.38
**Eosinophil count/mm**^**3**^	NA	278.9 ± 114.5	1576 ± 250.5 ***
**IgE titers IU/mL**	NA	448 ± 206.2	1667 ± 675.2 ***
**Treatment with IFNβ** (%) Yes No	NA NA	100 0	50 50
**Score (EDSS)** Mean +/- SD	NA	1.75 ± 0.1853	0.3333 ± 0.188 ****
**Hemogram** (cell number x10^6^) Leukocytes Lymphocytes Monocytes Granulocytes	5.976 ± 0.218 1.718 ± 0.088 0.077 ± 0.018 4.181 ± 0.177	5.115 ± 0.340 ^#^ 1.026 ± 0.126 ^##^ 0.1536 ± 0.034 3.846 ± 0.220	5.89 ± 0.579 1.487 ± 0.186 ^###^ 0.1253 ± 0.028 4.277 ± 0.479

SD = standard deviation; yr = year, NA = No applicable

*** p< 0.001 HIMS vs MS

**** p< 0.0001 HIMS vs MS

^#^ p< 0.05 MS vs Control

^##^ p< 0.0001 MS vs Control

^###^ p< 0.05 HIMS vs MS

### Haematological analysis

Cell counts and varying levels of each white fraction was measured using an Abacus Junior Vet veterinary automated analyzer (Vienna, Austria).

### Peripheral blood mononuclear cells (PBMCs) isolation and characterization of TAM receptors expression by flow cytometry

PBMCs from patients and healthy volunteer controls were obtained by centrifugation as previously described [27,32]. Briefly, the anti-coagulated blood sample was first diluted ½ volume/volume and centrifuged at low speed to separate platelet-rich plasma (200g for 10 min). The PBMCs were isolated from the cellular fraction by density gradient centrifugation employing Ficoll Paque Plus (GE Healthcare, Marlborough, MA, USA) following the standard protocol. Washed PBMCs were cryopreserved in 90% fetal bovine serum (FBS, Thermofisher Scientific, Argentina) plus 10% dimethyl sulfoxide (Sigma Aldrich, St. Louis, MO, USA), and stored in liquid nitrogen until use. TYRO3, AXL, and MERTK expression were analyzed in CD11b^high^ monocytes, CD1c^high^ dendritic cells (DCs), and CD4^+^ T cells compartment from cryopreserved PBMCs. Briefly, cells were thawed and washed first with PBS followed by PBS plus 2% FBS. After washing, cells were blocked in PBS plus 5% FBS for 30 minutes on ice and then incubated with appropriate antibody combinations against human CD11b-PercP/Cy5.5 (clone M1/70), CD4-APC/Cy7 (clone OKT4), and CD1c-PE/Cy7 (clone L161) from BioLegend for 30 minutes on ice. Rabbit anti-human TYRO3 (Novus Biological), biotin-conjugated goat anti-human AXL, and APC anti-human MERTK mAb IgG1 (R&D Systems) were used to evaluate TAM receptor expression after fixation and permeabilization (Cytofix/Cytoperm Kit, BD Bioscience, San Diego, CA, USA). Donkey anti-rabbit AF488 and PE-Streptavidin (Biolegend) were used as detection signals for TYRO3 and AXL, respectively. Cells were acquired using a FACS Canto I (Becton Dickinson). All analysis was carried out with FlowJo software (Tree Star Inc.).

### In vitro expansion of Th-subsets and intracellular IL-17, IFN-γ, IL-10, IL-13 and IL-4 determination

PBMCs were cultured in 24-well plates and stimulated with coated anti-CD3 (1μg/mL) and soluble anti-CD28 (1μg/mL). Fresh complete medium (RPMI-1640 supplemented with 10% FCS, 1% penicillin-streptomycin) was added at day 7. After expansion, cells were harvested at days 7, 10, or 17 and were incubated in ionomycin (1μg/mL), PMA (50nM), and Golgi Stop (BD Biosciences) at 37°C for 4 hours (h). Cells were washed with PBS, stained for viable cells with Zombie Violet 421 (Fixable Viability Dye), and fixed with Cytofix (BD Biosciences). Cells were permeabilized and incubated with anti-CD4-PE/Cy7 along with polyclonal rabbit anti-GAS6 or anti-PROS1-FITC for all subsets while anti-IFNγ-Alexa Fluor647, anti-IL-17-PE, anti-IL-10-PE, anti-IL-13-PE, and anti-IL-4-PE were utilized for each Th-subset, respectively. Donkey anti-rabbit AF647 were used as secondary antibody for GAS6 determination. Data were acquired using FACS Canto I (BD Biosciences) and analyzed using FlowJo software (Tree Star Inc.)

### Factor analysis of mixed data

In order to study the similarities between the MS and HIMS cohorts and the relationships with qualitative and quantitative variables, dimensionality reduction using factor analysis mixed data (FAMD) was performed. FAMD were computed in RStudio 3.5.3 using missMDA and FactoMineR packages. First NA missing values were imputed and categorical and continuous variables were detected automatically. Eigenvalues were then visualized, and the percentage of variances was explained by each principal component. Graphics and clustering analysis were performed using factoextra and autoEDA packages.

### In vitro differentiation of monocyte-derived DCs

Monocyte-derived DCs were generated from PBMCs as previously described [32]. CD14^+^ monocytes were isolated from PBMCs using the EasySep Human CD14 positive selection kit (StemCell Tech, Vancouver, Canada) following manufacturer’s instructions. 1 x 10^6^ Isolated CD14^+^ cells were cultured in a 6-well plate with 1.5 mL of complete RPMI medium, 300 U/mL of IL-4 (R&D Systems), 450 U/mL of GMCSF (R&D Systems) at 37°C and 5% CO2. Cytokine supplementation was performed again on day 3 by adding 1 mL RPMI-1640 supplemented with 10% FCS, 1% penicillin-streptomycin, 900 U/mL of IL-4 and 1350 U/mL GM-CSF. Monocyte-derived DCs were harvested on day 7. Recombinant cytokines were purchased from R&D Systems (Minneapolis, MN, USA).

### Mixed lymphocyte reaction (MLR) and GAS6 blocking

Human CD4^+^ T cells were obtained by negative selection (STEMCELL, USA) and co-cultured with human monocyte derived-DCs generated as described above at a 5:1 ratio for 72 h. When indicated, anti-GAS6 blocking antibody and the corresponding isotype control (R&D Systems) were added on day 0 of co-culture. On day 3, co-stimulatory markers on DC were evaluated by FACS. The activation markers were characterized by cell surface staining employing the appropriate combination of directly conjugated antibodies against human CD11c-PerCP/Cy5.5 (clone 3.9; BioLegend), HLA-DR-FITC (Clone L243; BioLegend), CD80-PE (Clone 2D10; BioLegend), CD40-PE/Cy7 (Clone 5C3; BioLegend) and CD86-biotin (Clone IT2.2; BioLegend) in combination with DyLight 649-conjugated streptavidin (BioLegend). Viability was assessed with Fixable Viability Dye eFluor 780 (e-Bioscience). After washing, cells were fixed with Cytofix/Cytoperm kit (BD Bioscience, San Diego, CA, USA). Cells were acquired using a FACS Canto I cytometer (Becton Dickinson), and all analysis was carried out with FlowJo software (Tree Star, Inc.).

### Quantitative PCR (qPCR)

For gene expression analysis, sorted CD4^+^ T cells (negative selection) were stimulated with 1μg/mL of plate-bound anti-CD3 and 1μg/mL soluble anti-CD28 (Life Technologies, Carlsbad, CA, USA) for 5 days. When indicated, 1 μg/mL of purified Annexin V (Biolegend) or 2 μg/mL anti-GAS6 blocking antibody and the corresponding isotype control (R&D Systems) were added on day 0 of co-culture. Treatment with 50 nM of recombinant human GAS6 (R&D Systems) was also performed every 2 days during 5 days when indicated. Cells were washed and then harvested with Trizol (Life Technologies, Carlsbad, CA, USA) following manufacturer’s instructions. Reverse transcription was performed using 100 ng of RNA in 20 μL of reaction volume by employing iScript cDNA synthesis kit (Bio-Rad, Hercules, CA, USA). Real time PCR reactions were assessed using 1 μL of cDNA in 10 μL of reaction volume by employing SsoAdvanced universal SYBR Green mix and CFX-Connect equipment (Bio-Rad, Hercules, CA, USA). Primers used in this study are listed in supplementary **S1 Table**. The reaction was normalized to housekeeping gene expression levels, and the specificity of the amplified products was checked through analysis of dissociation curves. To generate heat maps, qCT values obtained by 2ΔCt method were normalized to reference values (non-activated control or isotype conditions) and transformed by log10 function to scale up intergenic expression differences. Numbers inside colored squares are the mean value from each group in a given condition.

### Statistical analysis

Data are expressed as mean ± SEM. The Shapiro-Wilk test was used to define state normality and equal variance. Significant differences were determined by one-way analysis of variance (ANOVA) followed by Fisher multiple comparison test. Two-tailed, or one-tailed when indicated, unpaired Student´s t test was used to compare two groups. All statistical descriptions and number of donors used in each experiment are described in the respective figure legends. Statistical significance was set at *p* <0.05. The analysis was performed using GraphPad Prism software.

## Results

### Helminth infection and type II environment set differential TAM receptors and ligands expression pattern in patients with MS

The demographic and clinical characteristics of the 3 clinical groups are summarized in **Table 1**. The natural helminth-infected multiple sclerosis cohort (HIMS) shows significantly higher eosinophilia (1576±250.5 count/mm^3^, p<0.001) and plasma IgE titers (1667±675.2 IU/ml, p<0.001) compared with the uninfected MS cohort (278.9±114.5 count/mm^3^ and 448±206.2 IU/ml, respectively), confirming the presence of a type II environment response. On the other hand, a significant reduction of the total leukocyte number, mainly due to decreased circulating lymphocytes, was observed in the MS cohort compared with the HIMS (p<0.05) or healthy control (HC) groups (p<0.001), **Table 1**.

**Fig 1:**
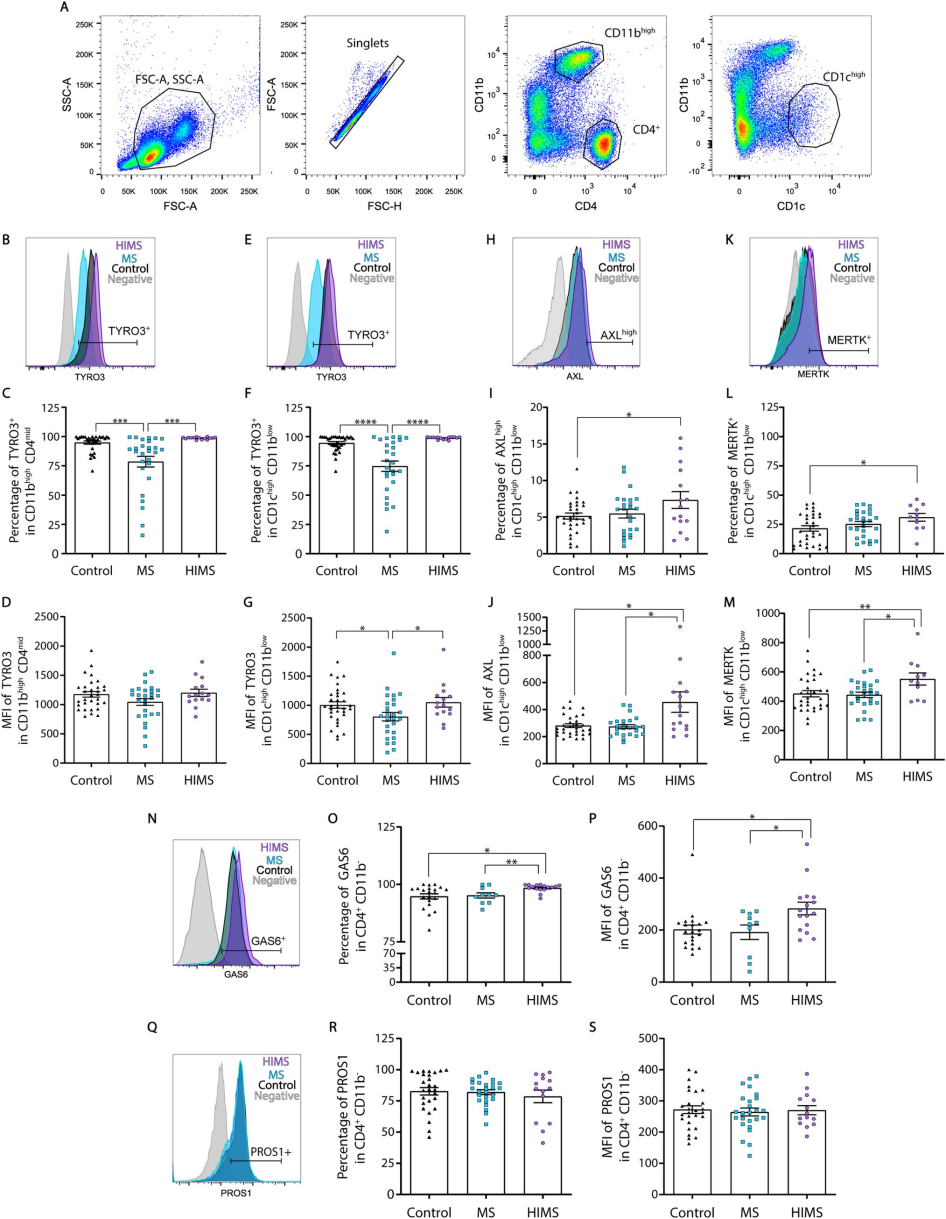
Helminth infection differentially modulates TAM receptor and their ligands expression in patients with MS and type 2 environment. **A**) Representative dot plots showing the three main sub-populations of mononuclear cells based on the expression of CD11b, CD4, and CD1c. TYRO3, AXL, and MERTK expression were evaluated in circulating monocytes (CD11b^high^CD4^mid^) and DCs (CD1c^high^CD11b^low^) of patients with MS, patients with HIMS, and healthy controls by flow cytometry. **B-G**) The percentage of TYRO3-positive as well as the mean fluorescent intensity (MFI) of the receptor on monocytes (**B, C, and D**) and DCs (**E, F, and G**) are graphed. **H, I, and J**) The percentage of AXL^high^ as well as the MFI of the receptor on DCs are shown. **K, L, and M**) The expression of MERTK on DCs is shown as both percentage and MFI. **N, O, and P**) The percentage of circulating CD4^+^ T cell expressing GAS6 as well as its MFI are shown. **Q, R, and S**) The percentage of circulating CD4^+^ T cell expressing PROS1 and its MFI are shown. Data is presented as a pool of independent samples included in the specific staining (Control N = 21–31; MS N = 10–27; and HIMS N = 11–16). One-way ANOVA with a Fisher post hoc test was performed to determine statistical significances, *p<0.05 **p≤0.01 ****p≤0.001. MS = multiple sclerosis, HIMS = helminth-infected multiple sclerosis.

TYRO3, AXL, and MERTK expression profiles were analyzed in circulating leukocytes as shown by the gating strategy in **Fig 1A**. We have found a significantly lower percentage (%) of monocytes (CD11b^high^CD4^mid^) and DCs (CD1c^high^CD11b^low^) expressing TYRO3 in patients with MS compared to HIMS or HC groups (**Fig 1B, 1C, 1E and 1F, respectively**). The mean expression level of TYRO3 per cell (indicated as MFI) did not show statistical differences in monocytes, but this receptor showed a significantly higher level in DCs of patients with HIMS or HC compared to MS (**Fig 1D–1G**). Intriguingly, we also observed an increased expression of TYRO3 in CD4^+^ T cells of patients from the HIMS cohort (**S1A Fig**), demonstrating that type-2 immunity has a broad effect on TYRO3. The concomitant helminth infection in patients with MS also increased the expression level of AXL in CD1c^high^ DCs, and notably, there were more cells (%) expressing high levels of AXL in the HIMS group (**Fig 1H–1J**). Additionally, the expression level of MERTK was also increased in DC of HIMS cohort (**Fig 1K–1M**). MERTK and AXL are not commonly expressed on CD4^+^ T cells; however, we found a clear dichotomy in their expression levels, with CD4^+^ T cells from the HIMS cohort expressing MERTK and CD4^+^ T cells from the MS cohort expressing AXL (**S1B and S1C Fig**). When we analyzed the levels of the two known TAM receptor ligands, GAS6 and PROS1, in circulating CD4^+^CD11b^-^ T cells, we found a significant increase of GAS6 level as well as the % of CD4^+^ T cell expressing GAS6 (**Fig 1N–1P**), but not differences in PROS1 expression in patients with HIMS compared with MS or HC (**Fig 1Q–1S**). Additionally, CD11b^high^CD4^low^ monocytes also showed a significant increase in GAS6 and PROS1 in the HIMS cohort compared with the MS cohort (**S1D and S1E Fig**), which could contribute to minimizing the pathological inflammation.

### Concomitant helminth infection induces a significantly lower percentage of Th17 cells and transcriptional program in patients with MS

**Fig 2:**
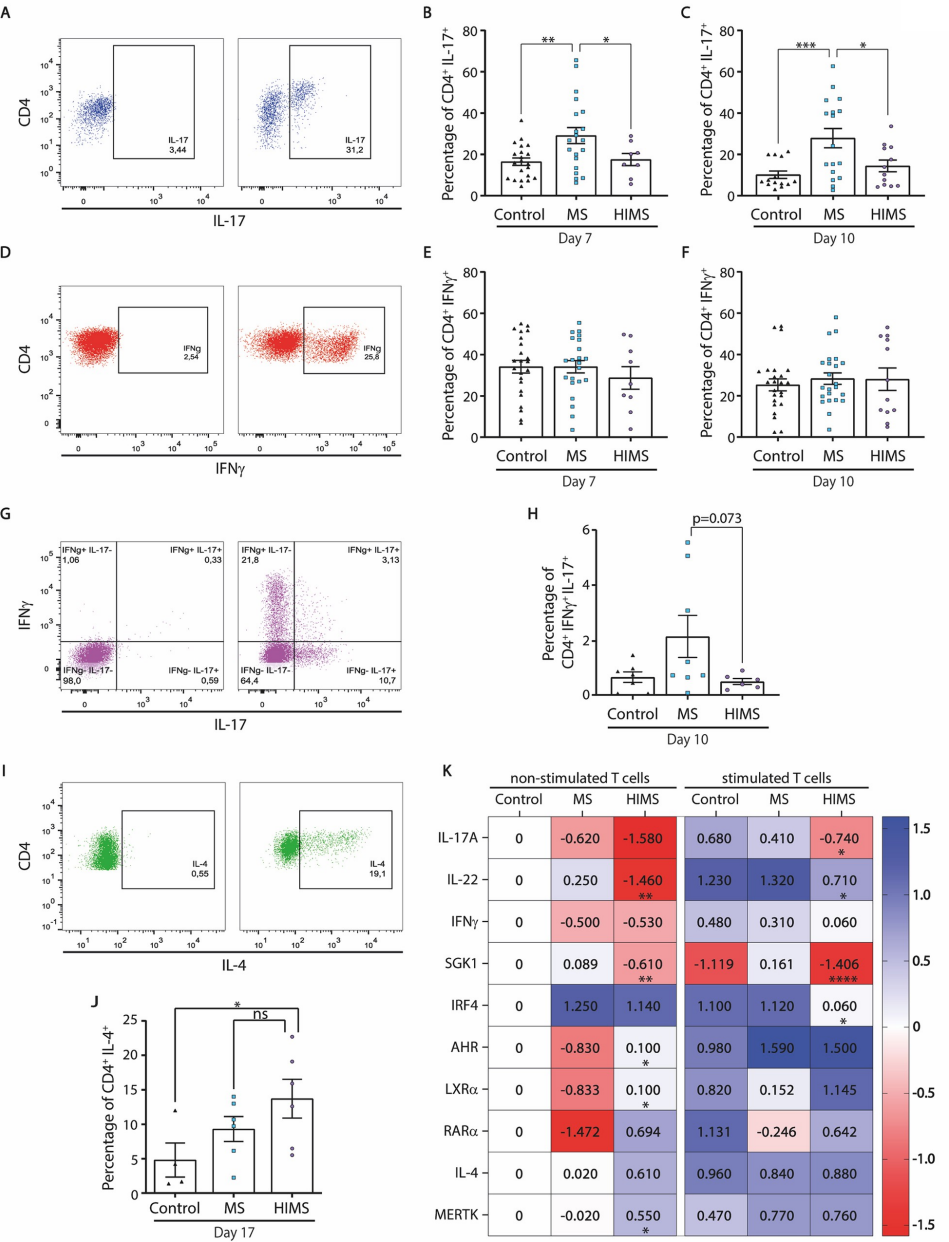
Concomitant helminth infection induced a significantly lower percentage of Th17 cells and transcriptional program in patients with MS. **A**) Representative dot plot showing CD4^+^IL-17^+^ cells, based on the isotype control gating on the left. **B and C**) The percentage of double positive cells at day 7 and 10, post activation with anti-CD3/CD28, are graphed. **D**) Representative dot plot showing CD4^+^IFNγ^+^ gate, based on the isotype control. **E and F**) The percentage of double positive cells after 7, and 10 days post-stimulation, are shown. **G and H**) Representative dot plot and the percentage of IL-17^+^IFNγ^+^ double positive in CD4^+^ T cells at day 10 after stimulation in the three clinical groups are shown. **I and J**) Representative dot plot showing the percentage of CD4^+^IL-4^+^ T cells, based on the isotype control, after 17 days post-stimulation, and the independent samples analyzed. **K**) Heat map showing relative mRNA expression profile of Th17 lineage-specific and related genes in stimulated vs non-stimulated T cells of MS and HIMS cohorts. All relative expression referenced the non-stimulated healthy control for basal expression levels (equal 0). The expression levels of indicated gene were evaluated by qPCR and referred as 2^dCT. *EF1A1* was used as the housekeeping gene. The numbers in the heat map indicate the mean value of at least 4 independent samples. Data is presented as a pool of independent samples included in the specific assay (Control N = 4–23; MS N = 6–22; and HIMS N = 4–12). One-way ANOVA with a Fisher post hoc test was performed to determine statistical significance, *p<0.05 **p≤0.01 ****p≤0.001. MS = multiple sclerosis, HIMS = helminth-infected multiple sclerosis.

To analyze which CD4^+^ T-helper subsets are expanded after activation, PBMCs were stimulated with anti-CD3 and anti-CD28, and intracellular IL-17^+^ (Th17), IFN-γ^+^ (Th1), and IL13^+^ or IL-4^+^ (Th2) staining was assessed at days 7, 10, and 17. The cytokine expression kinetics was first determined using PBMCs from controls subjects, shown in **S2A Fig**. The Th17 subset was significantly expanded in the MS cohort, but unlike these uninfected patients, the HIMS cohort failed to expand this subset at days 7 and 10 after activation (**Fig 2A–2C**). We did not find significant differences in the percentage of IFN-γ^+^CD4^+^ T cells among clinical groups at the same time points (**Fig 2D–2F**). However, a tendency of higher percentage of double-positive IFN-γ^+^IL-17^+^ T cells was found in the MS cohort (p = 0.0734) vs HIMS at day 10 **(Fig 2G and 2H)**. Surprisingly, the MS cohort also showed an expansion of IL-13^+^CD4^+^ T cells after activation (**S2B Fig**) as previously reported by Ochi et al [33]. With regards to IL-4-producing CD4^+^ T cells, an evident expansion of the Th2-subset was observed in the HIMS cohort even though the assay was performed with a lower number of samples (**Fig 2I–2J**). To better understand if each clinical group promotes a differential transcriptional pattern in sorted CD4^+^ T cells, we analyzed a panel of genes involved in the Th17 transcriptional program, cytokines, and negative regulatory feedback. We used the unstimulated HC group as reference and compared activated vs non-activated cells within and between MS and HIMS groups (**Fig 2K**). Interestingly, a differential gene expression pattern with higher *AHR*, *RAR-α*, *LXR-α* and *MERTK* and decreased *SGK1*, *IL-22*, and *IL-17* transcripts were observed in the HIMS group when compared with the MS group at the basal level. Furthermore, the prominent Th17 profile (*IL-17*, *IL-22*, *SGK1*, and *IRF4*) was expanded in the MS cohort after activation. In contrast, the HIMS group showed a lower expression of the transcriptional hallmarks of Th17 cells upon activation. Similarly, we observed a lower, but not statistically significant, level of *IFN-γ* in the HIMS group when compared with the MS group.

### Factor analysis of mixed data shows that the differential expression of TAM components explains and contributes to the segregation of both cohorts of patients

**Fig 3:**
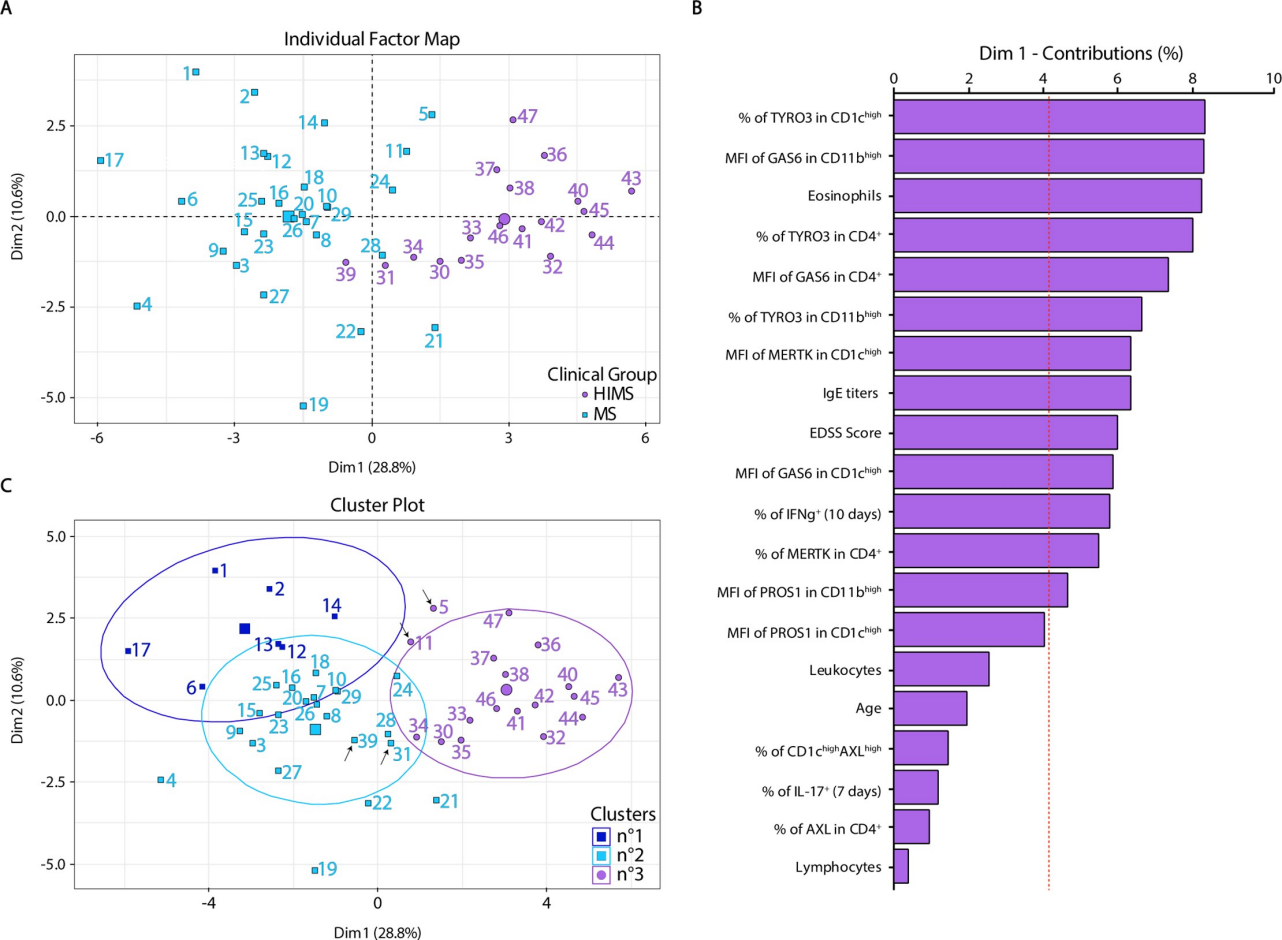
Factor analysis of mixed data shows that the differential expression of TAM components explains and contributes to the segregation of both cohorts of patients. To analyze the similarity and relationship among 24 variables studied in both MS and HIMS cohorts, we have performed the factor analysis of mixed data (FAMD) dimensionality reduction approach. We have included TAM receptors and ligands expression in each leukocyte population (CD11b^high^, CD1c^high^, and CD4^+^), the percentage of T-helper cells, score disease (EDSS), age, eosinophils, IgE titers, and total leukocyte and lymphocyte numbers. **A**) Individual Factor Map shows the distribution of the two most informative dimensions (Dim1 and Dim2) that explain the 39.4% of variance. Patients from MS group were highlighted in light-blue (1–29) and HIMS cohort (30–47) in purple for visualization. **B**) The bar graph indicates the top 20 individual variables that contribute to segregate Dim 1. The red dashed line indicates the expected average contribution. Variables over the cut-off would be considered as important contributors. **C**) Hierarchical clustering analysis shows that the cluster n°3 separately groups most of HIMS patients while cluster n°2 groups the majority of MS patients. The arrowheads show some patients of MS (5 and 11) or HIMS (31 and 39) clustering differentially.

In order to correlate the differential expression of the TAM axis with other clinical variables, we performed a factor analysis of mixed data (FAMD) dimensionality reduction approach grouping the expression of TAM receptors and ligands of each leukocyte population (CD11b^high^, CD1c^high^, and CD4^+^) with the T-helper subsets, disability score, age, eosinophils, IgE titer, as well as total leukocyte and lymphocyte numbers. The distribution map shows how the major informative dimensions (Dim1 and Dim2) explain most of the variance and separately segregate patients with MS from those with HIMS **(Fig 3A)**. The individual contribution of each variable to dimensions indicates that TYRO3 and MERTK expression in CD1c^high^ DCs, and GAS6 ligand on CD4^+^ T cells and monocytes CD11b^high^ are responsible for most of the variability for Dim1 and are strongly associated with the segregation of the HIMS cohort **(Fig 3B)**. When we performed a hierarchical clustering analysis to reveal the interaction among variables and patients, we observed that almost 100% of the HIMS cohort merged in one cluster while the MS cohort can be segregated to at least two clusters (**Fig 3C**), indicating a more complex interaction among components and patients. The variables that are over the expected average contribution (indicated as a dashed line) are considered as important contributors, and they could be useful for future analysis.

### CD4^+^ T cells from the HIMS cohort promote enhanced negative feedback on DCs that is dependent on the GAS6/TAM axis

**Fig 4:**
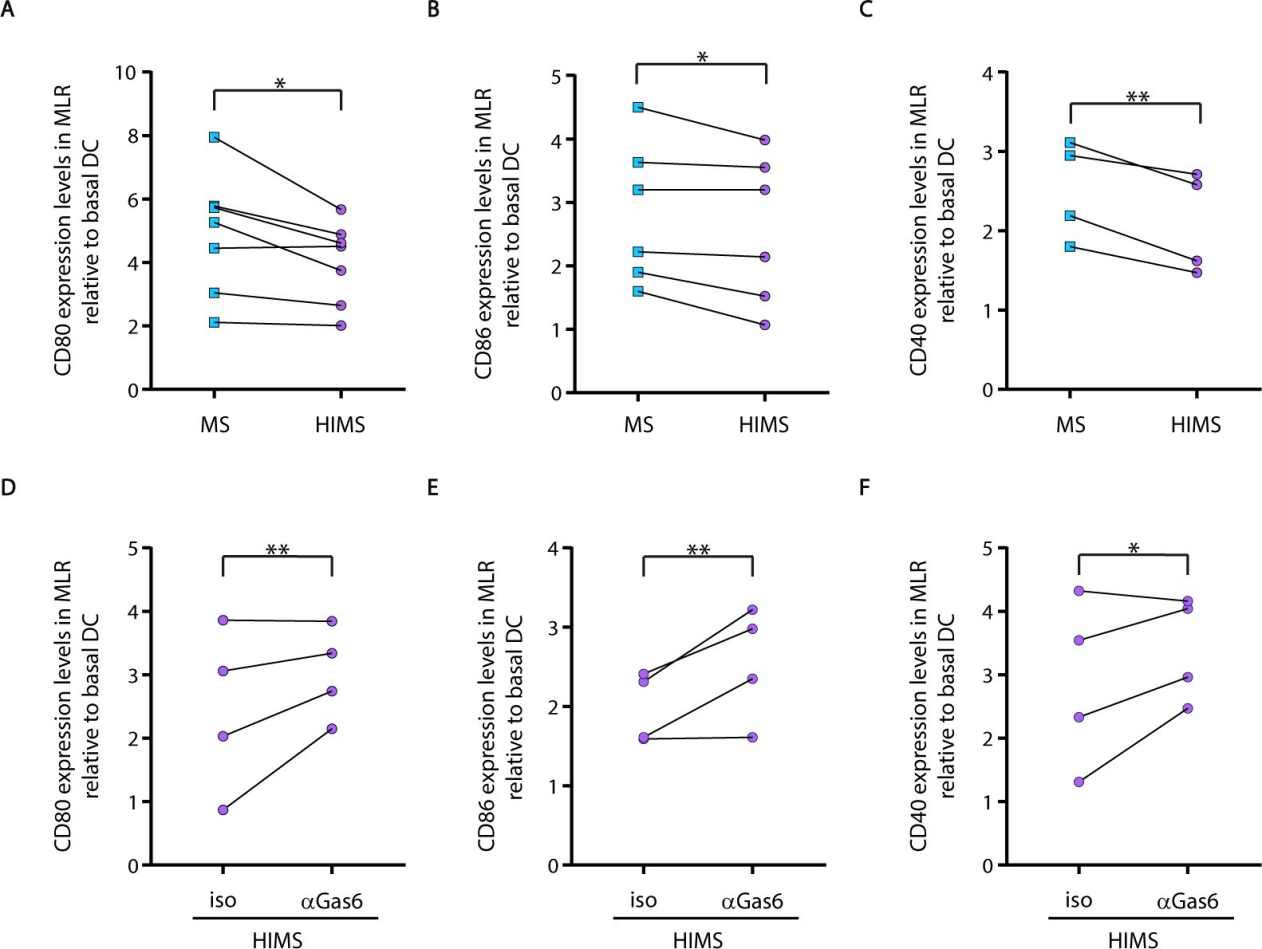
CD4^+^ T cells from patients with MS and concomitant helminth infection induced lower DCs activation that is GAS6-dependent. Mixed lymphocyte reaction (MLR) assay was performed by co-culturing monocyte-derived DCs from healthy controls with heterologous sorted CD4^+^ T cells from patients with MS and HIMS at a 1:5 ratio for 72 hours. Activation status of DC was evaluated by measuring surface levels of co-stimulatory molecules. **A, B, and C**) Relative expression levels of CD80 (**A**), CD86 (**B**), and CD40 (**C**) on CD11c^+^ DC cells after MLR assay. Expression levels were calculated relative to basal expression of DCs alone. **D, E, and F**) Blocking antibody against GAS6 (2 μg/mL) or its corresponding isotype was added in the MLR assays, and relative expression levels of CD80 (**D**), CD86 (**E**), and CD40 (**F**) on CD11c^+^ cells was calculated. The MLR assay was assessed employing 4–7 independent monocyte-derived DCs co-cultured with sorted CD4^+^ T cells from at least 4–6 different patients of each clinical group. Paired t-test was performed for each activation marker and statistical significances are indicated as *p<0.05 **p≤0.01. MS = multiple sclerosis, HIMS = helminth-infected multiple sclerosis.

Negative feedback from adaptive to innate immunity, particularly at the level of the T cell-DC interface, has emerged as a powerful mechanism to control the magnitude of the immune response [27]. Therefore, we compared the activation profile of DCs when they were challenged with sorted CD4^+^ T cells (1:5 ratio) from patients with MS, patients with HIMS, and HC, respectively, in a MLR assay as previously reported [27]. DCs were differentiated from CD14^+^ monocytes of HC for 7 days and then co-cultured with sorted heterologous CD4^+^ T cells from each clinical group for 72 hours. CD4^+^ T cells from patients with HIMS induced lower levels of the co-stimulatory molecules CD80, CD86, and CD40 on DCs when compared with the levels induced by CD4^+^ T cells from patients with MS (**Fig 4A–4C**). We did not detect significant differences in HLA-DR expression among clinical groups. These results highlight that CD4^+^ T cells from patients with HIMS actively engage immune-regulatory pathways.

Since GAS6 was increased in CD4^+^ T cells at basal levels in patients with HIMS, and could be an important contributor for the negative feedback at the T-cell-DC interface, we blocked this ligand in an MLR assay. Our results showed that under GAS6 blockade, CD4^+^ T cells from patients with HIMS enhanced the expression of the three co-stimulatory molecules CD80, CD86, and CD40 on DCs (**Fig 4D–4F**), indicating the critical role of this ligand as the negative feedback signal for this cohort. The anti-GAS6 treatment has differentially impacted co-stimulatory markers when using CD4^+^ T cells from HC and MS groups (**S3A–S3C Fig**).

### GAS6 expression is increased in CD4^+^IL-10^+^ in comparison with CD4^+^IL-17^+^ cells of the HIMS cohort and restrains the development of the Th17 subset

**Fig 5:**
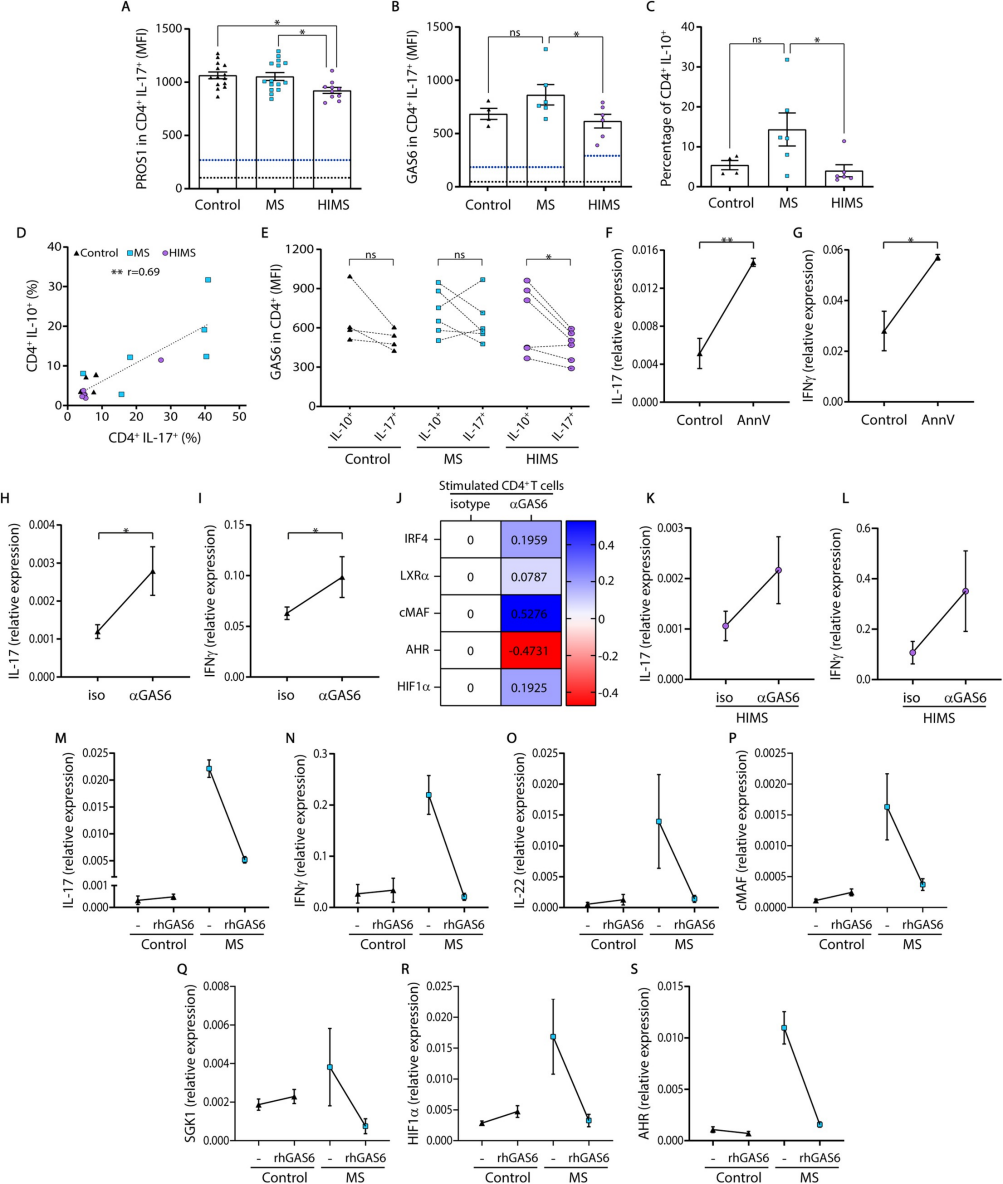
GAS6 expression is higher in CD4^+^ IL-10^+^ cells than in CD4^+^IL17^+^ cells and modulates Th17 development in HIMS. **A and B**) Expression levels of PROS1 and GAS6 in CD4^+^ IL-17-expressing cells, measured as MFI, are shown. Independent data for each specific staining are shown, and the negative threshold is indicated as a black dash line. The baseline expression of non-stimulated CD4^+^ T cells is indicated as a blue dash line. **C and D**) The percentage of CD4^+^IL10^+^ cells was positively correlated with the percentage of Th17 subset. **E**) Comparison of GAS6 expression levels between IL-10^+^ and IL17^+^ CD4^+^ T cells within each clinical group. **F-I**) Sorted CD4^+^ T cells from healthy controls were activated and expanded with anti-CD3/CD28 during 5 days in the presence of AnnV (1 μg/mL), a competitor for phosphatidylserine binding with TAM ligands, or blocking anti-GAS6 (2 μg/mL) antibody; IL-17 and IFNγ mRNA expression were evaluated by quantitative PCR (qPCR). **J**) Transcription factor genes related to the Th17 subset (*IRF4*, *LXRα*, *cMAF*, *AHR and HIF1α*) were also evaluated in stimulated CD4^+^ T cells under anti-GAS6 treatment by qPCR. **K and L**) mRNA level of IL-17 and IFNγ were evaluated in activated CD4^+^ T cells from HIMS group after blocking GAS6. **M-S**) CD4^+^ T cells from controls and patients with MS were activated with anti-CD3/CD28 and treated with 50 nM of recombinant human GAS6 every 2 days during 5 days and IL-17 and IFNγ, IL-22, cMAF, SGK1, HIF1α, and AHR were evaluated by qPCR. Correlation was assessed by Spearman test. Flow cytometry data is presented as a pool of independent samples included in the specific staining (Control N = 4–14; MS = 6–15; HIMS = 6–10). qPCR was performed with at least 5 independent HC, 3 of MS, and 3 of HIMS donors. One-way ANOVA with a Fisher post hoc test was used in A-D and statistical significances are indicated as *p<0.05 **p≤0.01. One-tailed paired t-test was used to compare blocking conditions. MS = multiple sclerosis, HIMS = helminth-infected multiple sclerosis.

Immune pathology frequently arises when critical checkpoints fail to be engaged during the inflammatory response, inducing unbalanced activation. We found first that both PROS1 and GAS6 ligands were upregulated after activation, compared to the baseline of non-stimulated CD4^+^ T cells, independent of the cohorts (**S4A and S4B Fig**). Intriguingly, patients with HIMS condition showed higher levels of GAS6 in acutely isolated CD4^+^ T cells, but after 10 days post-activation, the MS group maintained higher levels of both ligands in the Th17 subset compared with the HIMS cohort (**Fig 5A and 5B**). Similarly, CD4^+^IL-10^+^ cells were also increased and positively correlated with the Th17 subset (**Fig 5C and 5D,** r = 0.69; p<0.01 and **S4C Fig**) within the MS cohort, which denotes robust compensatory mechanisms that are insufficient at controlling the pathogenic response in these patients. Interestingly, when comparing GAS6 expression between CD4^+^IL-10^+^ and Th17 cells, a significantly higher GAS6 levels in the IL-10^+^ than IL-17^+^ cells was observed only in the HIMS group, denoting that the balance of this ligand between these effector cells is critical to keep pathological cells under control (**Fig 5E**). The TAM ligands require binding to PtdSer and cellular contact in order to activate the TAM receptors, and we have recently shown that activated T cells transiently expose this phospholipid on their surface (27). To test if this mechanism has an active role in Th17 induction, a PtdSer-binding competitor, Annexin V (AnnV) was added during CD4 stimulation and expansion. The number of clustering cells was reduced in the presence of AnnV during the first 3 days of culture; however, it promoted an increased expression of CD44, higher percentage of CD4^+^ IL-17^+^ cells after 7 days of activation (**S4D and S4E Fig**), as well as increased IL-17 and IFN-γ RNA transcripts at day 5 (**Fig 5F and 5G)**. Similarly, the use of a GAS6 blocking antibody increased the mRNA level of IL-17, IFN-γ, cMAF, HIF1-α, and IRF4 in activated CD4^+^ T cells from HC (**Fig 5H–5J**). Furthermore, blocking GAS6 in activated CD4^+^ T cells from the HIMS group also led to an increased expression of IL-17 and IFN-γ (**Fig 5K and 5L**). In the same sense, treatment with 50nM of hrGAS6 dramatically reduced not only IL-17 and IFN-γ, but also IL-22, cMAF, SGK-1, HIF1-α and AHR expression in activated CD4^+^ T cells from patients with MS (**Fig 5M–5S respectively**). These results suggest that GAS6 and PtdSer binding are critical signals to control not only T cell activation but also of IFN-γ and Th17 expansion in a cell contact-dependent manner. Altogether, our results suggest that GAS6 tempers not only an innate immune response but also regulates Th17 development in an autocrine/paracrine way.

## Discussion

Multiple factors may be responsible for, or contribute to, the increased incidence and prevalence of MS in recent decades globally [7]. However, since these population changes have occurred over a rather short period of time, genetic factors are an unlikely cause, indicating that the environment strongly influences MS risk. One of these environmental factors is known as the biome depletion theory, which states that the loss of species diversity from the ecosystem of the human body in modern industrialized countries leads to immune dysregulation and a subsequent increase in the prevalence of chronic inflammatory-associated diseases [34]. This paradigm appreciates the importance of an array of microbes and helminths as essential for immune system development and regulation [34,35]. In support of this theory, Correale et al. have previously shown that well-tolerated natural helminth infection protect against worsening of MS symptoms in humans. Moreover, since anti-parasitic treatment results in the worsening of helminth-infection symptoms, its use leads to a significant increase in clinical and radiological MS activities, an increase in the number of IFN-γ and IL-12 producing cells, and a reduction in regulatory signals after three months of anti-helminths treatment [23]. Type 2 immune responses are elicited to clear parasitic helminth infections; however, helminths have also evolved and acquired a variety of immunotolerant regulatory mechanisms that manipulate immune development and function to ensure their survival [36,37]. Deciphering these regulatory mechanisms may provide new therapeutic insights for allergic and autoimmune disorders, metabolic syndrome, and chronic low-grade inflammation [38]. Even though numerous studies support helminth therapy as a promising concept [16,34,38], we still need a better understanding of the dominant immunological mechanisms and the types of helminths that can be used for therapy, which will require additional preclinical studies as well as systematic clinical trials.

**Fig 6:**
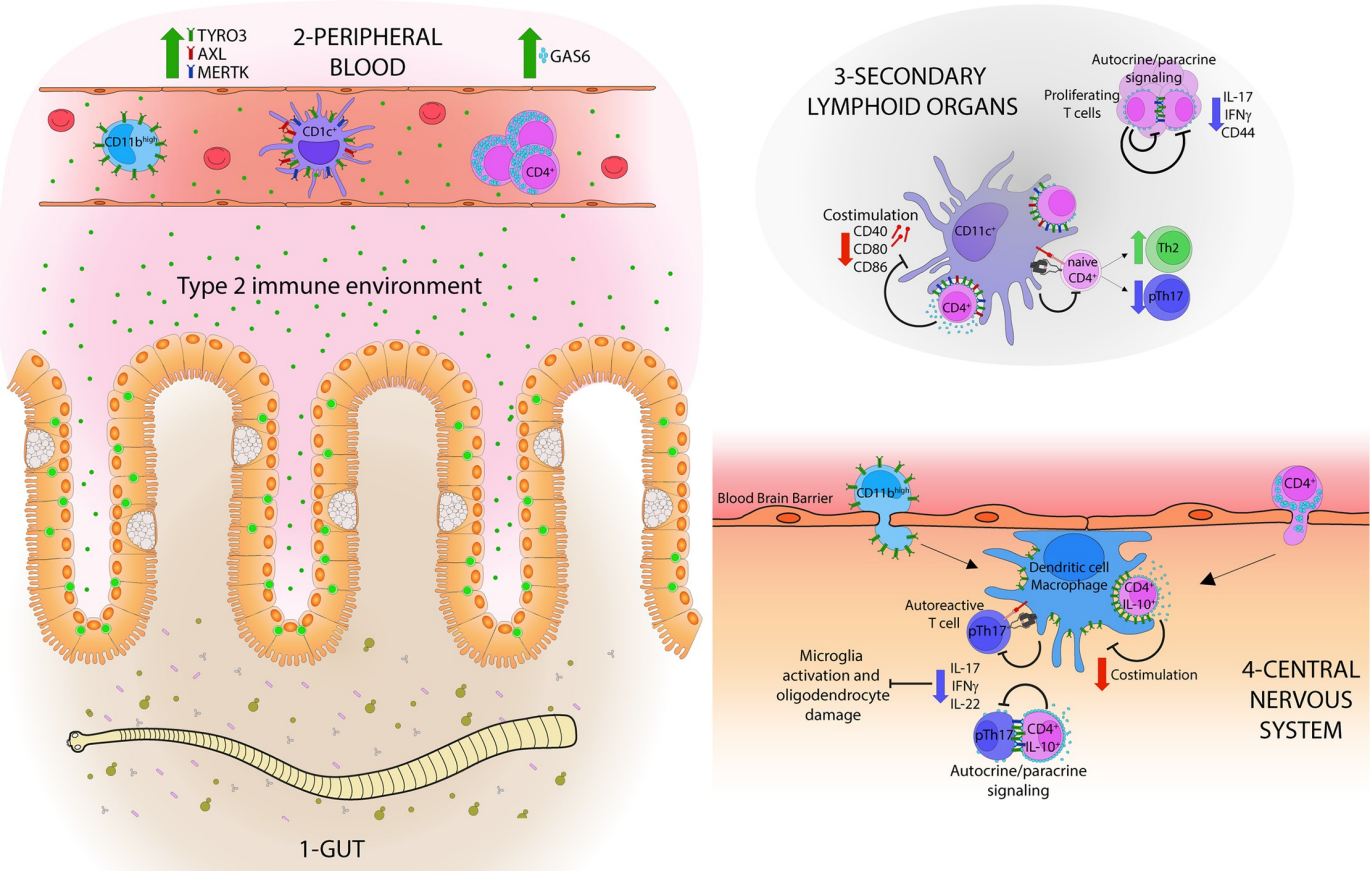
Helminth-induced type 2 immunity enhances regulatory TAM/GAS6 signaling to dampen Th17 pathological responses in multiple sclerosis. The host type 2 immune response is elicited to clear parasitic helminth infections; however, the co-evolution of these parasites has promoted a variety of mechanisms to oppose and redirect immune responses by manipulating immune cell programming and function. Integrating our results with the current knowledge, we propose that gastrointestinal (**1**) helminths infections can enhance the regulatory axis of TAM receptors (TYRO3, AXL, and MERTK) in peripheral blood (**2**) CD11b^high^ and CD1c^high^ cells, and their ligand GAS6 in CD4^+^ T cells of patients with MS. This negative regulatory pathway could be essential for dampening co-stimulatory signals (CD40, CD80, and CD86) of antigen-presenting cells and controlling the pathological Th17 (pTh17) response at secondary lymphoid organs (**3**). The active chronic infection resets T helper response toward Th2/regulatory signals, limiting pathological Th17 (pTh17) effector response. This reprogramming of CD4^+^ T cells and GAS6 signaling under a helminth-induced type 2 environment could be essential for maintaining the balance between CD4^+^ IL-10^+^ and Th17 cells and reducing inflammatory IL-17 and IFNγ signals in the central nervous system (**4**). In summary, GAS6 tempers not only the innate immune response but also regulates Th17 development in an autocrine/paracrine manner.

In the present work, we showed how natural helminth infections enhanced the negative regulatory axis of TAM receptors and their ligands in patients with MS and could be essential for controlling the inflammatory response. We have previously observed that TYRO3 is increased on human monocyte-derived DC of helminth-infected subjects [28]. Here, we showed that parasite infection primes innate and adaptive immune cells with an increased expression of the TAM axis. Schistosome eggs-derived antigen triggers a tolerogenic response by decreasing CD40, CD80, and CD86 co-stimulatory markers on DCs [39], and our findings reveal that CD4^+^ T cell from patients with HIMS also induced lower DC activation in a GAS6-dependent manner. We postulate that the helminth-type 2 environment hijack the TAM signaling to temper innate immune response and consequently reduce the development of pathogenic Th1/Th17 responses (**Fig 6**, proposed model). The multivariate analysis supports the relevance of the TAM axis since most of the variability could be explained by the differential expression of TYRO3, GAS6, and MERTK in innate immune cells to discriminate between the HIMS and MS cohorts.

Emerging data on Th17-mediated diseases suggest that different types of IL-17-producing T cells exist in vivo, and these can be divided into pathogenic or non-pathogenic Th17 cells depending on the transcriptional program and functional properties [40,41]. Furthermore, the plasticity of this subset is evident in chronic and pathological immune responses as well as resolution [42]. The ability of parasites to defy host immunity reflects their masterful manipulation of the immune system by affecting CD4^+^ T cell differentiation, Treg cell induction, B cell isotype switching, and Breg cell induction [16], which in turn determine the effector cell responses of the host. Here, we reported that the HIMS condition resets CD4+ T cells programming and redirects the immune response by increasing expression of IL-4, RAR-α, AHR, LXR-α and MERTK along with decreased SGK1, IL-22, and IL-17 when compared with the MS cohort. These results support the hypothesis that controlled helminths coexistence enhances negative regulatory circuits to mitigate the inflammatory response. The number of patients associated with a particular species of helminth in this study is too low for a conclusive correlation between helminth species and modulatory effect. Nonetheless, the species listed in this report could be useful for the design and planning of future studies using controlled helminth infection in patients with MS (**S2 Table**). Identifying the helminth species or their products for future therapy trials is the major challenge. Specific eligibility criteria need to be addressed when evaluating prospective therapy, such as feasible domestication, controlled exposure to population, and positive cost/benefit ratio [16,34].

Previously we have shown that T cell-derived PROS1 from healthy volunteer samples play a critical role in the negative regulation of DC activation [27]. Despite structural homology between GAS6 and PROS1, both ligands have distinct affinities for the TAM receptors and are also differentially expressed in various cell types [43]. The functions of GAS6 seem to be limited to those caused by activation of the TAM receptors while PROS1 has both TAM receptor-dependent and independent activities [24]. GAS6 plays a critical role as an immunoregulatory signal at the interface of innate and adaptive immunity, and our findings suggest that the balance of this ligand between regulatory and effector cells is critical to restrain pathological Th17 cells, as observed under HIMS condition. Conversely, the higher inflammatory milieu in the MS cohort keeps high numbers of IL-17^+^ and IL10^+^ but without contraction, supporting the idea that true suppressor CD4^+^IL10^+^ also depends on GAS6 signaling. When GAS6 was neutralized during expansion of the Th17 subset, we found increased expression levels of IL-17, IFN-γ, cMAF, IRF4, and HIF1-α. In the same sense, the treatment with recombinant GAS6 reduced the transcript levels of IFN-γ and the signature of pathogenic Th17 cells induced by CD4^+^ T cells from MS group. These results demonstrate the role of GAS6 in controlling Th17 expansion and the transcriptional program and plasticity of the Th17 subset (**Fig 6**, proposed model).

Limitations of this study included the transversal analysis and limited cell numbers. The transversal analysis was only capable of providing a snapshot of the dynamic immune response and pathological course. The cell numbers recovered from each sample varied across patients and precluded the performance of all assays for every recruited patient.

In agreement with our results, it has been shown that GAS6 enhances the suppressive activity of Tregs in vitro and in vivo [44]. Furthermore, the exogenous administration of GAS6 into the brain of wild-type mice had a therapeutic effect in improving the recovery from damage after cuprizone withdrawal along with a beneficial effect on the clearance of cellular and myelin debris, promoting remyelination and maturation of oligodendrocyte progenitor cells [45]. On the other hand, loss of GAS6 and AXL signaling while undergoing cuprizone challenge results in extensive axonal damage, prolonged neuroinflammation, severe demyelination, a greater reduction in oligodendrocytes number, and an overactivation of microglia and motor deficits [46]. When MOG-induced experimental autoimmune encephalomyelitis was performed in Gas6−/− mice, worse clinical scores, delayed recovery from damage, a higher expression of pro-inflammatory molecules, and a significant increase of macrophages infiltration were observed. In contrast, the direct intracerebral delivery of GAS6 is protective with evidence of less demyelination and enhanced remyelination relative to controls [47]. Interestingly, a recent study also showed that the intra-articular adenoviral delivery of TAM agonists reduced joint pathology in a murine model of rheumatoid arthritis [48]. Therefore, GAS6-TAM signaling is a key modulator not only for the homeostatic immune response but also to promote the remyelination process, though this has only been shown in animal models. Even though very few studies are available, autopsy studies on patients with MS revealed that soluble AXL and MERTK can act as decoy receptors and block GAS6 binding, resulting in dysregulation of protective GAS6-mediated signaling, leading to prolonged lesion activity [49].

In conclusion, our work substantiates the hypothesis that enhancing the TAM axis in a manner similar to helminth infection could be a promising treatment for autoimmune diseases by mitigating the detrimental inflammatory response and empowering regulatory signals to restore tissue homeostasis.

## Supporting information

S1 Table List of Primers sequences. (DOCX)

S2 Table Number of Patients with MS and natural helminth infection discriminated by spp of helminth and IFN-β treatment. (DOCX)

S1 Fig Differential TAM expression in CD4^+^ population and GAS6 and PROS1 in CD11b^high^ monocytes of patients with HIMS compared with patients with MS. **A-C)** TYRO3 and MERTK are preponderantly expressed in CD4^+^ T cells of HIMS while AXL-expressing lymphocytes are increased in MS patients. **D-E)** GAS6 and PROS1 expression in CD11b^high^CD4^mid^ monocytes are graphed as MFI relative to isotype. Data is presented as a pool of all independent samples included in a specific staining of each assay (Control N = 21–31; MS = 11–27; HIMS = 11–16). One-way ANOVA with a Fisher post hoc test was performed to determine statistical significances, *p<0.05 **p≤0.01 ***p≤0.005 ****p≤0.001. MS = multiple sclerosis, HIMS = helminth-infected multiple sclerosis, MFI = Mean Fluorescence intensity.
(TIFF)

S2 Fig Kinetics of intracellular cytokine production by CD4^+^ T cells after TCR stimulation. **A)** The intracellular production of IL-17, IFNγ, and IL-4 in CD4^+^ T cells was determined by stimulating PBMCs from healthy subjects with 1ug/mL of plate bound anti-CD3 and soluble anti-CD28 at different time points. To analyze the level of intracellular cytokines, cells were harvested at 7, 10 and 17 days post stimulation plus a reboost with ionomycin and PMA for the last 4 hours before harvesting. Viability staining with Fixable viability dye AF450 was included. **B)** The percentage of IL-13^+^ cells in CD4^+^ T lymphocytes after 10 days of stimulation in the three clinical groups are shown (Control N = 13; MS = 16; HIMS = 7). The threshold for the positive signal of cytokines was determined with the corresponding isotype. One-way ANOVA with a Fisher post hoc test was performed to determine statistical significances. MS = multiple sclerosis, HIMS = helminth-infected multiple sclerosis.
(TIFF)

S3 Fig GAS6 neutralization enhances CD80 but not CD86 expression on DCs after MLR assay. Mixed lymphocyte reaction (MLR) assay was performed by co-culturing monocyte-derived DCs from HCs with heterologous sorted CD4^+^ T cells from patients with MS or HCs at a 1:5 ratio during 72 h. The activation status of DCs was evaluated by measuring surface levels of co-stimulatory molecules. **A-C**) Relative expression levels of CD80 **(A)**, CD86 **(B)** and CD40 **(C)** on CD11c^+^ referred to that of DCs alone. Blocking antibody against GAS6 (2 ug/mL) or its corresponding isotype were used. The MLR assay was assessed employing 4 independent monocyte-derived DCs co-cultured with sorted CD4^+^ T cells from at least 4 different donors of each group. Paired t-test was performed for each activation marker and statistical significances are indicated as *p<0.05 **p≤0.01. MS = multiple sclerosis, Control = Healthy control, HC = healthy control.
(TIFF)

S4 Fig PROS1 and GAS6 expression in activated CD4^+^ T cells, and how blocking PtdSer leads to increased CD44 expression and IL-17 production. **A-B**) The levels of PROS1 and GAS6 were evaluated on total CD4^+^ T cells after 7 days post-stimulation (Control N = 4–19; MS = 6–23; HIMS = 6–10). The negative threshold is indicated as a black dash line. The basal expression of non-stimulated CD4^+^ T cells is indicated as blue dash line. **C**) Representative dot plot showing the percentage of CD4^+^ IL-10^+^ T cells compared to the isotype control after 10 days post-stimulation. **D-E**) The activation status of CD4^+^ T cells in the presence of AnnV (1 ug/ml), a competitor for PtdSer binding with TAM ligands, was evaluated in control PBMCs stimulated with 1ug/mL of anti-CD3 and anti-CD28 after 7 days (N = 4). **D)** CD44 levels on CD4^+^ cells and **E)** intracellular levels of IL-17 were determined by flow cytometry. The expression levels of CD44 and IL-17 are referred to fluorescent minus one as negative signal. Annexin V was added from day 0 of stimulation and at day 3 of culture. One-way ANOVA with a Fisher post hoc test was performed to determine statistical significances. Paired t-test was performed for D and E, and statistical significances are indicated as *p<0.05.
(TIFF)
