# Supplementary material for: GAS6 signaling tempers Th17 development in patients with multiple sclerosis and helminth infection
Source: PLoS Pathog. 2020 Dec 21;16(12):e1009176. doi: 10.1371/journal.ppat.1009176 (PMC7785232; doi:10.1371/journal.ppat.1009176)
Supplement: S1 Table — (DOCX) [file ppat.1009176.s001.docx]

**S1 Table**. List of Primers sequences

| Eef1A1 Fwd | TCGGGCAAGTCCACCACTAC |
| --- | --- |
| Eef1A1 Rv | CCAAGACCCAGGCATACTTGA |
| hMER Fwd | CTCTGGCGTAGAGCTATCACT |
| hMER Rv | AGGCTGGGTTGGTGAAAACA |
| hRAR alpha Fwd | GCCTGGACATCCTGATCCTG |
| hRAR alpha Rv | TCCGCACGTAGACCTTTAGC |
| hAHR Fwd | AACATCACCTACGCCAGTCG |
| hAHR Rv | CAAAGCCATTCAGAGCCTGT |
| hIL17 Fw | TCCCACGAAATCCAGGATGC |
| hIL17 Rv | TGTTCAGGTTGACCATCACAGT |
| hIFNg Fw | ACTGACTTGAATGTCCAACGCA |
| hIFNg Rv | ATCTGACTCCTTTTTCGCTTCC |
| hIL4 Fw | CCAACTGCTTCCCCCTCTG |
| hIL4 Rv | TCTGTTACGGTCAACTCGGTG |
| hIRF4 Fwd | TGACAACGCCTTACCCTTCG |
| hIRF4 Rv | CCTGTCACCTGGCAACCATTT |
| hSGK1 Fwd | CATATTATGTCGGAGCGGAATGT |
| hSGK1 Rv | TGTCAGCAGTCTGGAAAGAGA |
| hNR1H3 (LXR-a) Fwd | GGAGGTACAACCCTGGGAGT |
| hNR1H3 (LXRa) Rv | AGCAATGAGCAAGGCAAACT |
| hIL-22 Fwd | CGGAGTCAGTATGAGTGAGCG |
| hIL-22 Rv | TCTAGCAGGGAAAGGGGGTT |
| hcMAF Fwd | CTGTGTATGGGGGCTGACTT |
| hcMAF Rv | GGTGTGCTAGGGGAAGATGA |
| hHIF1-a Fwd | ACTAGCCGAGGAAGAACTATGAA |
| hHIF1-a Rv | TACCCACACTGAGGTTGGTTA |
