# Supplementary material for: GAS6 signaling tempers Th17 development in patients with multiple sclerosis and helminth infection
Source: PLoS Pathog. 2020 Dec 21;16(12):e1009176. doi: 10.1371/journal.ppat.1009176 (PMC7785232; doi:10.1371/journal.ppat.1009176)

**S2 Table**: Number of Patients with MS and natural helminth infection discriminated by spp of helminth and IFN-β treatment. Pie distribution is also shown

| **Nº of Patients with** | **No Treatment** | **IFN-β Treatment** |
| --- | --- | --- |
| Strongyloides stercolaris | 3 | 1 |
| Ascaris lumbricoides | 1 | 3 |
| Enterobius vermicularis | 1 | 1 |
| Hymenolepis nana | 3 | 0 |
| Trichuris trichura | 1 | 2 |
| Ancylostoma duodenale | 0 | 2 |


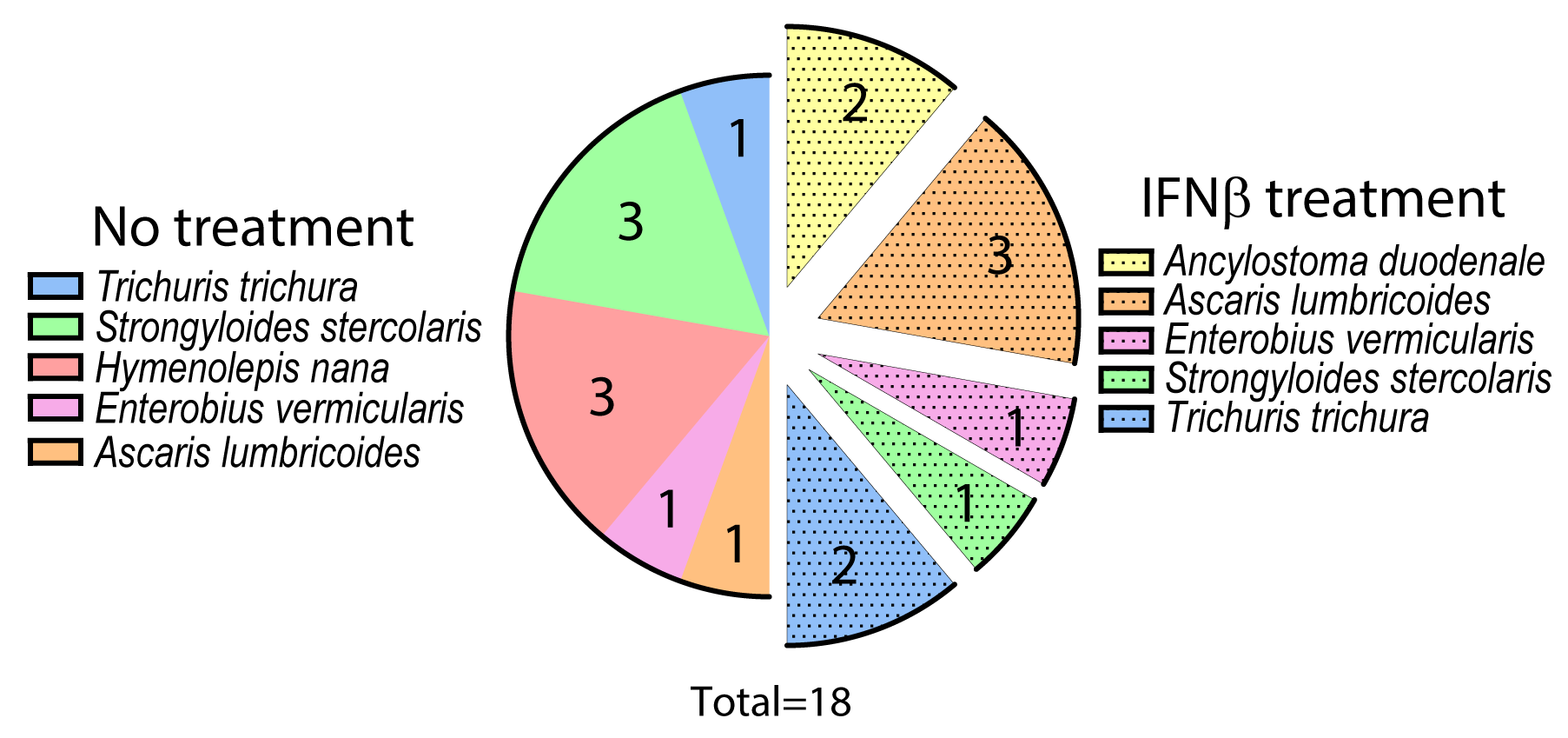

Supplement: S2 Table — (DOCX) [file ppat.1009176.s002.docx]
