# Supplementary figures and images for: GAS6 signaling tempers Th17 development in patients with multiple sclerosis and helminth infection
Source: PLoS Pathog. 2020 Dec 21;16(12):e1009176. doi: 10.1371/journal.ppat.1009176 (PMC7785232; doi:10.1371/journal.ppat.1009176)

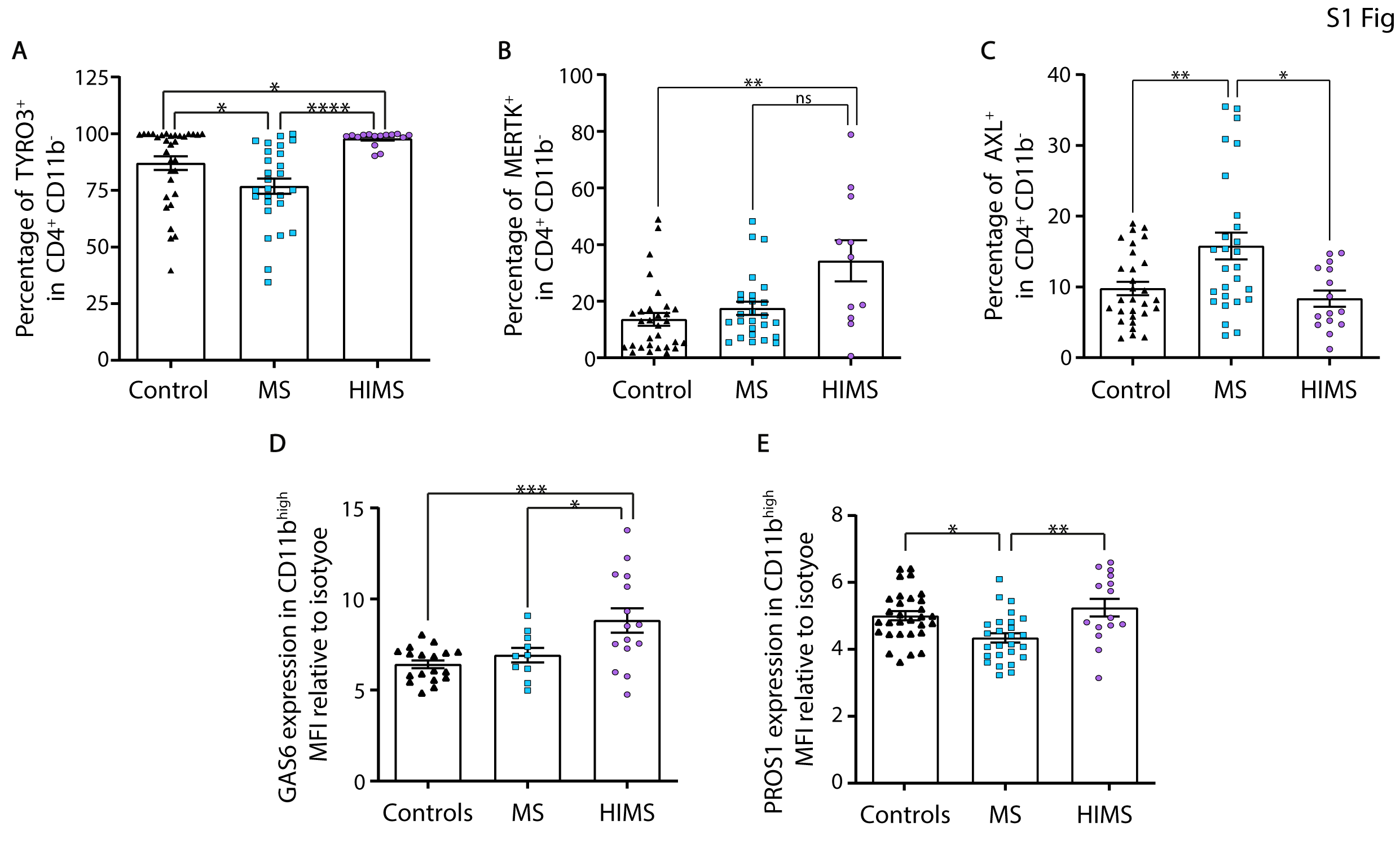

Supplement: S1 Fig — A-C) TYRO3 and MERTK are preponderantly expressed in CD4+ T cells of HIMS while AXL-expressing lymphocytes are increased in MS patients. D-E) GAS6 and PROS1 expression in CD11bhighCD4mid monocytes are graphed as MFI relative to isotype. Data is presented as a pool of all independent samples included in a specific staining of each assay (Control N = 21–31; MS = 11–27; HIMS = 11–16). One-way ANOVA with a Fisher post hoc test was performed to determine statistical significances, *p<0.05 **p≤0.01 ***p≤0.005 ****p≤0.001. MS = multiple sclerosis, HIMS = helminth-infected multiple sclerosis, MFI = Mean Fluorescence intensity. (TIFF) [file ppat.1009176.s003.tiff]

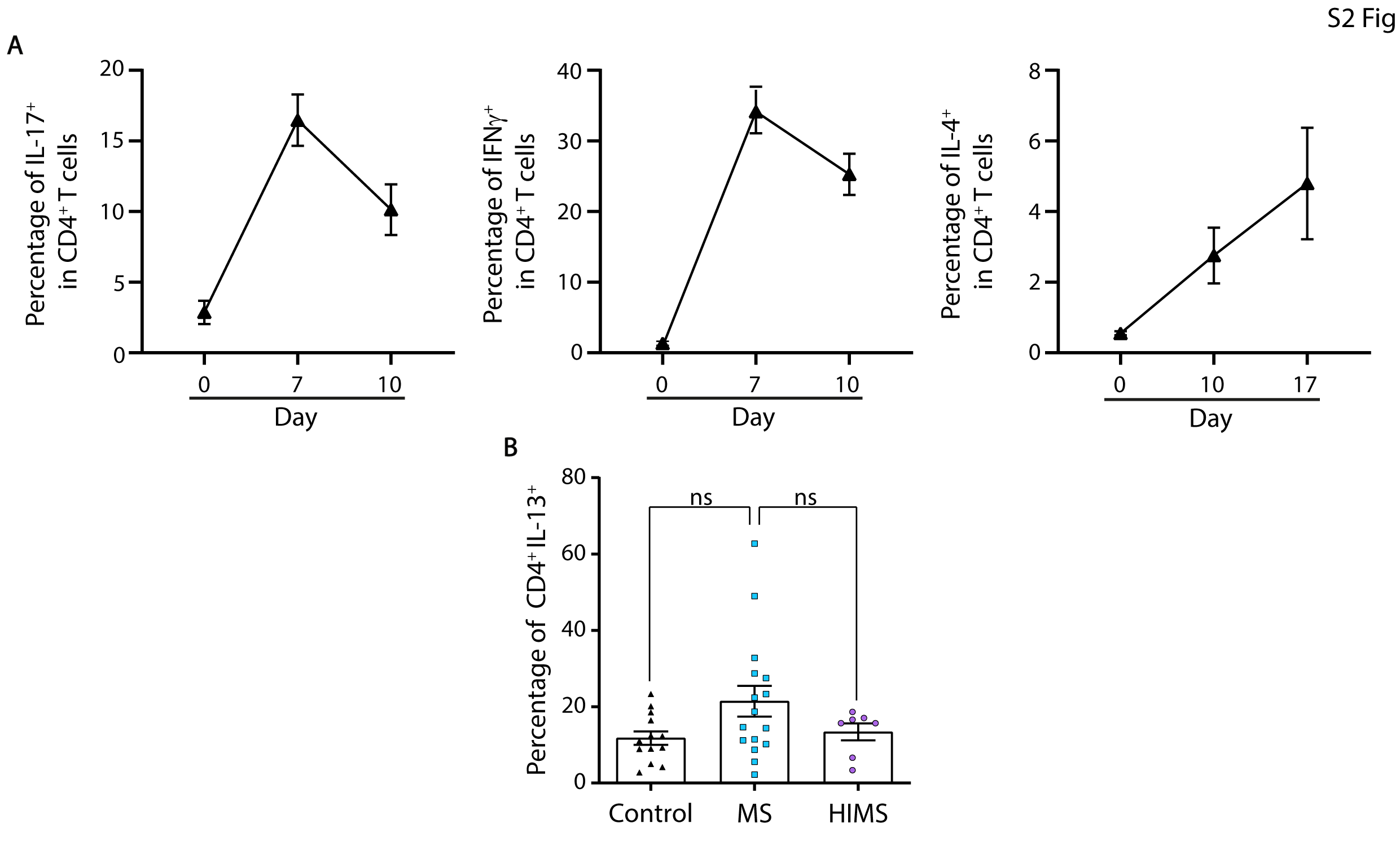

Supplement: S2 Fig — A) The intracellular production of IL-17, IFNγ, and IL-4 in CD4+ T cells was determined by stimulating PBMCs from healthy subjects with 1ug/mL of plate bound anti-CD3 and soluble anti-CD28 at different time points. To analyze the level of intracellular cytokines, cells were harvested at 7, 10 and 17 days post stimulation plus a reboost with ionomycin and PMA for the last 4 hours before harvesting. Viability staining with Fixable viability dye AF450 was included. B) The percentage of IL-13+ cells in CD4+ T lymphocytes after 10 days of stimulation in the three clinical groups are shown (Control N = 13; MS = 16; HIMS = 7). The threshold for the positive signal of cytokines was determined with the corresponding isotype. One-way ANOVA with a Fisher post hoc test was performed to determine statistical significances. MS = multiple sclerosis, HIMS = helminth-infected multiple sclerosis. (TIFF) [file ppat.1009176.s004.tiff]

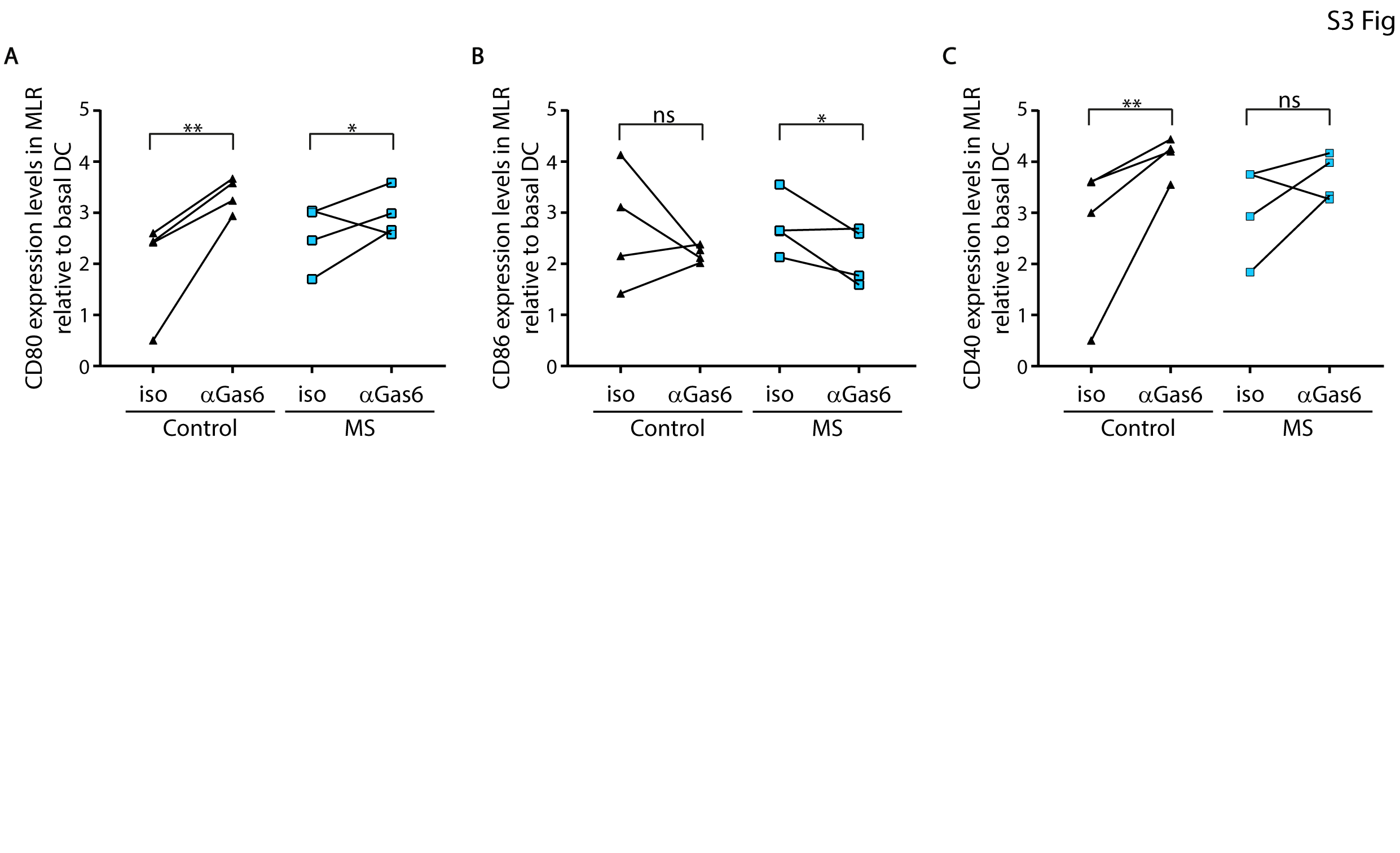

Supplement: S3 Fig — Mixed lymphocyte reaction (MLR) assay was performed by co-culturing monocyte-derived DCs from HCs with heterologous sorted CD4+ T cells from patients with MS or HCs at a 1:5 ratio during 72 h. The activation status of DCs was evaluated by measuring surface levels of co-stimulatory molecules. A-C) Relative expression levels of CD80 (A), CD86 (B) and CD40 (C) on CD11c+ referred to that of DCs alone. Blocking antibody against GAS6 (2 ug/mL) or its corresponding isotype were used. The MLR assay was assessed employing 4 independent monocyte-derived DCs co-cultured with sorted CD4+ T cells from at least 4 different donors of each group. Paired t-test was performed for each activation marker and statistical significances are indicated as *p<0.05 **p≤0.01. MS = multiple sclerosis, Control = Healthy control, HC = healthy control. (TIFF) [file ppat.1009176.s005.tiff]

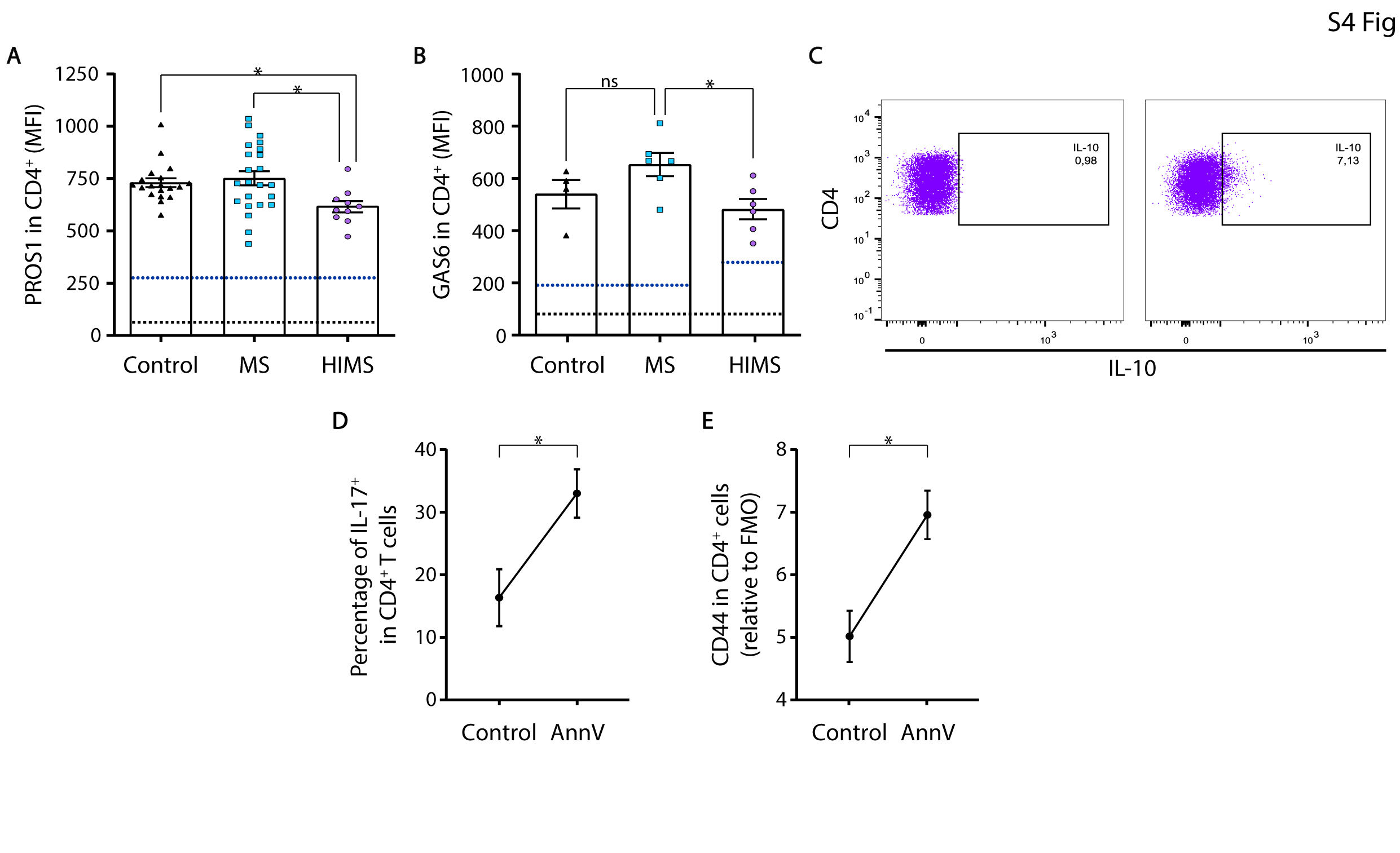

Supplement: S4 Fig — A-B) The levels of PROS1 and GAS6 were evaluated on total CD4+ T cells after 7 days post-stimulation (Control N = 4–19; MS = 6–23; HIMS = 6–10). The negative threshold is indicated as a black dash line. The basal expression of non-stimulated CD4+ T cells is indicated as blue dash line. C) Representative dot plot showing the percentage of CD4+ IL-10+ T cells compared to the isotype control after 10 days post-stimulation. D-E) The activation status of CD4+ T cells in the presence of AnnV (1 ug/ml), a competitor for PtdSer binding with TAM ligands, was evaluated in control PBMCs stimulated with 1ug/mL of anti-CD3 and anti-CD28 after 7 days (N = 4). D) CD44 levels on CD4+ cells and E) intracellular levels of IL-17 were determined by flow cytometry. The expression levels of CD44 and IL-17 are referred to fluorescent minus one as negative signal. Annexin V was added from day 0 of stimulation and at day 3 of culture. One-way ANOVA with a Fisher post hoc test was performed to determine statistical significances. Paired t-test was performed for D and E, and statistical significances are indicated as *p<0.05. (TIFF) [file ppat.1009176.s006.tiff]
